# Going off antiretroviral treatment in a closely monitored HIV “cure” trial: longitudinal assessments of acutely diagnosed trial participants and decliners

**DOI:** 10.1002/jia2.25260

**Published:** 2019-03-14

**Authors:** Gail E Henderson, Margaret Waltz, Karen Meagher, R Jean Cadigan, Thidarat Jupimai, Sinéad Isaacson, Nuchanart Q Ormsby, Donn J Colby, Eugène Kroon, Nittaya Phanuphak, Jintanat Ananworanich, Holly L Peay

**Affiliations:** ^1^ Social Medicine University of North Carolina at Chapel Hill Chapel Hill NC USA; ^2^ Biomedical Ethics Research Program Mayo Clinic Rochester MN USA; ^3^ Center of Excellence in Pediatric Infectious Diseases and Vaccines Chulalongkorn University Bangkok Thailand; ^4^ SEARCH, Thai Red Cross AIDS Research Centre Bangkok Thailand; ^5^ U.S. Military HIV Research Program Walter Reed Army Institute of Research Silver Spring MD USA; ^6^ Henry M. Jackson Foundation for the Advancement of Military Medicine Bethesda MD USA; ^7^ RTI International Research Triangle Park NC USA

**Keywords:** phase I clinical trials, HIV clinical trials, analytic treatment interruption, research ethics, informed consent, qualitative research

## Abstract

**Introduction:**

The South East Asia Research Collaboration in HIV (SEARCH) RV411 clinical trial in Thailand was a systematic investigation of analytic treatment interruption (ATI) in individuals diagnosed and treated since Fiebig stage I acute HIV infection. Here, we explore decision‐making processes and perceptions of trial participation in a phase I trial that raised important ethical considerations, to identify potential areas of improvement in this relatively new field of HIV research. Similar considerations apply to other HIV phase I trials, especially those involving ATI, making this trial a model to identify challenges and opportunities in promoting informed choice.

**Methods:**

Using longitudinal semi‐structured interviews and a validated questionnaire, we examined how decisions to join or decline the trial were made, whether there was evidence of decisional conflict, and reactions to the trial outcomes. We also explored contrasting views and experiences in this small trial cohort. We report analyses of data from these questionnaires and interviews, conducted from February through December of 2016 with the 14 SEARCH cohort participants who either joined (n = 8) or declined (n = 6) participation in RV411.

**Results:**

The eight participants and six decliners had low overall decisional conflict, which remained low over time. Decision making was more difficult for decliners than participants, at least initially. While all interviewees described being satisfied with their decisions, our study identified important negative consequences for a few individuals, including seroconversion, negative experiences with optional procedures and disappointment due to rapid viral rebound.

**Conclusions:**

Although our results reflect the experiences of a small group invited to join this trial, our overall finding of low decisional conflict even while some individuals reported negative experiences provides lessons for clinical trial investigators. We developed points‐to‐consider in helping participants make informed choices, to support participants during the trial and to support decliners in their decisions.

## 1 Introduction

In 2009, the South East Asia Research Collaboration in HIV (SEARCH) at the Thai Red Cross AIDS Research Centre began diagnosing and treating individuals with acute HIV infection 1, and inviting them to join SEARCH upon diagnosis. SEARCH 010 is a long‐term cohort study with frequent clinical follow‐up, antiretroviral treatment (ART) provision per US and Thai treatment guidelines and psychosocial support. The cohort now numbers over 500 participants, who may be recruited to sub‐studies that include HIV remission trials. In 2016, our decision‐making study (DMS) initiated with longitudinal interviews and questionnaires collected from cohort members recruited to remission trials. We aim to understand motivations to join or decline and whether, how, and why decision satisfaction may change over time 2, 3. The second “cure” trial recruited from the cohort was RV411, a phase I trial investigating whether early ART alone might facilitate control of HIV 4. Cohort members diagnosed in Fiebig stage I HIV infection and started on ART at a median of two weeks after estimated HIV acquisition were recruited. Fiebig I stage corresponds to the first two weeks following infection when HIV nucleic acid is detected in the absence of p24 antigen (viral capsid core protein) and HIV seroconversion 5. Viraemic control after analytic treatment interruption (ATI) was the study's primary endpoint.

Early‐phase trials typically offer no direct medical benefits and potentially significant risks, thus raising concerns about informed consent 6, 7, 8, 9. RV411 also included withdrawing treatment for individuals doing well on ART, a controversial practice given that standard treatment is lifelong ART 10. RV411 participants were informed that stopping ART was not a form of treatment and that no direct benefits were expected. The consent form described potential risks of ATI: acute retroviral syndrome (ARS), HIV drug resistance, increased risk of transmitting HIV to sexual partners, the possibility of exclusion from future trials, unknown longer‐term health effects of ATI, and the possibility of testing HIV‐antibody positive (seropositive) for “people who had previously tested negative (seronegative) by the standard anti‐HIV testing.” This latter risk, known as seroconversion, is particularly important in Thailand. In the Thai context, HIV serology testing is often required for employment, mortgage applications and private health insurance, thus raising the potential for social harm and discrimination as a result of a positive HIV test. As all RV411 participants were SEARCH cohort members, they each had a minimum of four additional years in which they could receive continued follow‐up in the clinical research cohort setting. This was explained to them in the informed consent process.

As a systematic investigation of treatment interruption in acutely treated individuals, RV411 offered the opportunity to explore decision making and perceptions of trial participation in the context of important ethical considerations. These are known and unknown risks of ART interruption, including risks to participants and to third parties outside the research, alongside an unlikelihood that participants will receive any direct medical benefits from the trial. Using longitudinal, semi‐structured interviews and questionnaires, our DMS examines how decisions to join or decline the trial were made, assesses decisional conflict, and explores reactions to the trial outcomes. Through analysis of individual perspectives and experiences, we identify cases that illustrate the complexity of decision making for both participants and decliners. Together, these data inform ethical implications and potential areas of improvement in this relatively new field of HIV research.

## 2 Methods

SEARCH cohort participants were approached sequentially for recruitment to RV411 after being identified as meeting strict enrolment criteria, including being on ART and virally suppressed for at least two years 4. Recruitment continued until the desired sample size of 8 participants was achieved. After receiving trial information, eight joined and six declined.

RV411 was conservatively designed with resumption of ART at first confirmed HIV viral load above 1000 copies/mL. The hypothesis that at least one participant might control the virus for 12 weeks (84 days) was disproved when all eight participants experienced viral rebound within 13 to 48 days (median 26 days) after ATI. After resuming treatment, their viral loads declined to less than 50 copies/mL by a median of 17 days. ATI did not result in ARS, HIV‐related symptoms, opportunistic infections, or new drug resistance mutations. However, of the six participants who had a negative fourth Generation HIV test result prior to ATI, four became seropositive after ATI. In a *Nature Medicine* publication, the investigators recommended that future remission trials with ATI should include additional interventions to boost immune capacity 4. Since that publication, two of the four who became seropositive reverted to testing seronegative by fourth Generation HIV test.

The 14 individuals were who were recruited for RV411 were all invited to join the DMS, which was presented as a separate study and required a separate informed consent process. All 14 continued their enrolment in the SEARCH cohort, which facilitated our ability to enroll the RV411 decliners as participants in the DMS study. An independent DMS researcher completed the DMS consent process and conducted the interviews. Interviewees were paid 500 Thai Baht (approximately 15 USD) per interview.

The DMS was approved by the Chulalongkorn University IRB in Thailand. IRBs at University of North Carolina at Chapel Hill, RTI International, and Walter Reed Army Institute of Research determined it exempt for US‐based investigators.

### 2.1 Semi‐structured interviews and decisional conflict scale

DMS RV411 trial participants were interviewed three times: (1) within five days of enrolment, (2) four weeks after ATI or as soon as viral rebound occurred and (3) at the trial's conclusion, 3 to 4 months after the last participant went back on ART. Decliners were interviewed twice: (1) within five days of declining, and (2) after the trial concluded. All received a brief summary of the trial outcome at the final interview.

Interviews began with completing the Decisional Conflict Scale 11. This validated scale measures certainty in decision making, incorporating feeling informed, clarity and support in decision making, and satisfaction with the choice 11 (see Appendix). Response categories span strongly agree to strongly disagree. Scoring is 0 (no decisional conflict) to 100 (extremely high decisional conflict). Scores of <25 have been associated with implementing decisions with little conflict, while scores >37.5 have been associated with decision delay and feeling unsure of the decision 12. The Cronbach's alpha was 0.97.

The first interview explored experiences with HIV diagnosis, trial expectations, trial decision making and advice to others considering a similar trial. Participants’ second interviews focused on trial experiences. At the time of the second interview, five [P01, P02, P03, P07, P08] had experienced viral rebound and had restarted ART, while three [P04, P05, P06] had not. The final interviews reflected on decisions to join or decline and advice to others considering a similar trial. Interview audio‐recordings were transcribed and translated from Thai to English and then checked for accuracy. Transcripts were coded using MAXQDA qualitative software 3. Six people coded the interviews, working in three pairs. One pair included a native Thai speaker who coded the original Thai version of the interview transcript and a native English speaker who coded the English translation. This pair compared their coding, and assessed whether codes could be applied the same way in the Thai and English versions. The six coders also met regularly to discuss and revise the codebook and definitions, and ensure thematic nuances were appropriately represented 3 One topic of discussion, for example, focused on differences between the concepts of altruism and reciprocity. Conventional context analysis was used 13, with attention to complexities in analysing longitudinal data, including comparing individual data over time and group data at several points in time 14, 15. In analysing and interpreting the qualitative and quantitative data, we paid attention to common themes regarding ease of decision making and trial experiences as well as reports of individuals whose experiences diverged.

## 3 Results

Table 1 shows RV411 participant and decliner demographics. They were predominantly male, with similar length of time since initiating ART. Decliners were somewhat older and better educated than participants.

**Table 1:** Demographics

	Participants (n = 8)	Decliners (n = 6)
Sex		
Male	7	5
Female	1	1
Age (median, range)	29.6 years (22.2 to 34.4 years)	33.2 years (26.9 to 48.4 years)
Education		
High school/basic technical school	3	
Advanced technical school	1	
Bachelor degree	3	6
Master degree or higher	1	
Time on antiretroviral treatment (median, range)	2.7 years (2.4 to 5.4 years)	2.6 years (2.3 to 5.4 years)

Information about perceptions of trial risks and benefits, from the first interviews, is summarized in Table 2. While both groups anticipated similar risks and benefits, they were valued differently by participants and decliners.

**Table 2:** Participant and decliner perceptions of RV411 benefits and risks at initial interview

Topic	Participants	Decliners
Close monitoring (requires time commitment of up to twice weekly)	Mainly a benefit	Mainly a burden
Treatment Interruption	Both risk and benefit Risk mitigated by close monitoring	Too risky Risk not adequately mitigated
Altruism/reciprocity	Strong motivation	Motivating but not sufficient to offset potential risk; some worry about not helping others

### 3.1 Why join? Perceptions of participants

The opportunity to stop ART motivated all participants. One imagined he would feel “normal,” and “be happy and live like when I did not have the virus…” [P05] Being off ART would eliminate the burden of remembering to take daily medication and the fear that being seen taking it would lead to disclosure. Understanding how their immune system would fare in the absence of medication was also intriguing: “Sometimes I want to challenge myself – what will happen if I stop ART?” [P02] Participants worried about the health impacts of taking ART long term and saw ATI as a way of giving their body “a break.” All participants described altruistic motivations, including advancing scientific research, contributing to the SEARCH effort, and helping others with HIV. Mutual benefit was important to many: “I wanted to know how strong my immunity is. I am infected with HIV and if there is research that allows other people to get benefits from my HIV status, I think it is ok.” [P05] Another, when asked to advise about future trials, said that “every trial has a risk. But if I can accept the risk and get benefits to myself or others then we should try.” [P06] Most described a reasonably easy decision to participate. One participant [P07] was not planning to join but changed his mind after being re‐contacted with additional information; he reported feeling influenced by the trial team.

When asked about risks of ATI, all mentioned at least one possible risk described in the consent form, particularly increased viral load and developing ART resistance; none mentioned heightened risk of transmission, although researchers emphasized and screened for this risk at each weekly clinic visit. Participants believed that close monitoring by the trusted SEARCH staff could protect them from trial risks. One stated that if he got too nervous about viral rebound, he could withdraw [P04]. Of the six who had not seroconverted prior to trial participation, one [P06] mistakenly thought he already had seroconverted; another [P05] did not know his sero‐status; two understood the risk of seroconversion but were reassured by trial monitoring and procedures [P01, P08]; and one [P04] acknowledged the possibility of seroconversion but expected it would happen one day regardless of his participation.

### 3.2 Why not join? Perceptions of decliners

Most decliners reported that the time commitment required by RV411 was too burdensome: they lived far away or could not get time off work. For many, the risks of participating were also too great. One [D05] stated, “There is no result from any research [suggesting] that you can stop the drug after only taking it for 2 to 3 years, even if you started the drug earlier [in the acute phase]. There is no guarantee… We are the first group, so I'd rather wait.” A similar risk calculus was reported by those doing well on ART and two who, despite qualifying for RV411, perceived they were not. One [D06] argued that ART permits him to live as he did before being diagnosed, without worry, so why interrupt his treatment? He also speculated that even if he stopped ART successfully, his viral load might rise after close monitoring ended, without his awareness, reflecting concern also mentioned by some participants about long‐term health consequences. In contrast, two [D04, D05] were worried that their underlying health made it too risky to stop ART. Two others were very concerned about remaining seronegative [D06, D01], although they also mentioned being bothered that their decision was “selfish.” When asked about potential trial benefits, most decliners cited similar possible benefits including positive aspects of stopping ART, feeling “normal,” and helping others. The decliner most worried about his health [D05], however, could not think of any potential benefit.

### 3.3 How did participants experience the RV411 trial?

For two participants, an ART holiday – no matter how short – lived up to expectations. One [P02] said, “I almost forgot what I am” [a person living with HIV]. The remaining six reported positive aspects but also at least one concern, including unexpected worry about their immune system while unprotected by ART, the burden of frequent monitoring visits, and optional procedures (five volunteered for at least one of inguinal lymph node biopsy, colon biopsy, lumbar puncture and leukapheresis). Several described invasive optional procedures as painful: one [P01] chose not to repeat a lymph node biopsy; another [P07] preferred not to repeat the lumbar puncture, but agreed anyway citing the importance of post‐ATI data. Lastly, there were negative experiences after seroconversion. One participant [P03] described being “shocked … I did not know what I should do. I walked around like I lost my mind … for a whole day. But now, I am ok.”

### 3.4 How were decisions about RV411 assessed at the final interviews?

At the start of the final interviews, the interviewer summarized the trial's results: (1) all participants experienced viral rebound; and (2) the study collected important scientific information directed to finding better remission strategies.

All interviewees described being satisfied with their decisions. Decliners felt confident they had made the right decision, and were not surprised by the trial outcome. Most participants said that even though they had to restart ART, they would make the same decision again because of the possibility for and actual perceived benefits to themselves and to science. Two who seroconverted [P08, P03], however, reported they might not make the same decision, or as one [P03] noted, at least secure his job first. Some participants expressed surprise that the trial ended so quickly and disappointment by the speed with which they, themselves, experienced viral rebound. One [P02] said: “[The result] is not that different from what I expected. I knew it [viral load] would increase … But mine increased rapidly. I felt it was very fast.”

Interviewees were asked in the first and last interviews what advice they would give others considering a trial with ATI. Decliners gave similar responses before and after the trial, stating that individuals needed to make their own decisions after learning the benefits and the risks. Participants’ advice changed somewhat over time. At the first interview, most advocated joining a similar trial because it would benefit themselves and others. After the trial ended, they were more cautious. For example, one participant [P04] initially stated that joining was better than lifelong ART, but after the trial was over, said: “I want them to think about the long run and consider whether it has more benefits than risks, or not.” Other participants still advised taking a risk to help others, as one [P05] reflected in his final interview: “If you can take a risk and accept it, you should give it a try. At least there will be a benefit to the research team. If it's successful, others will get benefits from our devotion.” Although participants’ advice to others became more nuanced, all participants and decliners reported being willing to consider participating in future trials through SEARCH.

### 3.5 What do quantitative decisional conflict scores reveal?

Consistent with the qualitative data, decisional conflict was low overall, especially for trial participants (Table 3). Participants’ mean scores were 12.6/100 at first and final interviews, and rose slightly at the second interview to 14.6/100. Decliners had higher decisional conflict at the first time point (mean score 28.0/100) with a reduction at the final interview (mean score 19.1/100).

**Table 3:** Decisional conflict mean total scores for RV411 participants and decliners

	First interview	Second interview	Final interview
Participants (n = 8)	12.6/100 (range 0 to 33.3)	14.6/100 (range 0 to 32.2)	12.6/100 (range 0 to 30)
Decliners (n = 6)	28.0/100 (range 7.8 to 55.6)		19.1/100 (range 0 to 36.7)

**Figure 1:**
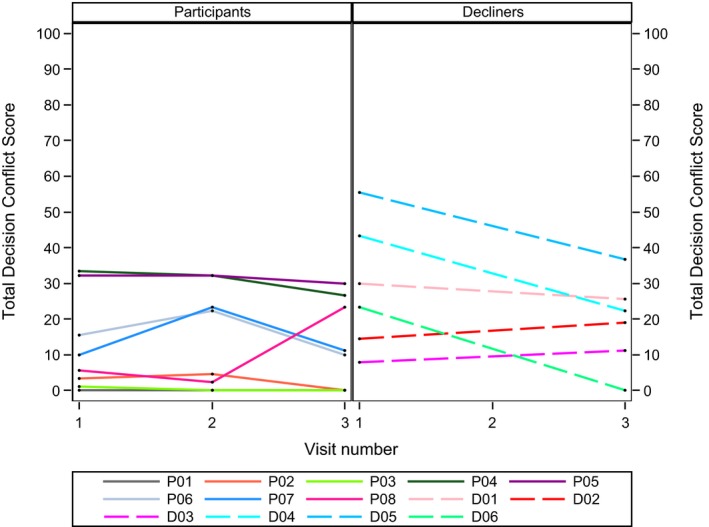
Decisional conflict scores for participants and decliners.

Figure 1 presents decisional conflict scores for each participant and decliner over time. Some participant scores start and stay low, while others increase at either the second or third interview. Decliner data are distributed across a wider range of scores at the first and last interview, but exhibit similar patterns: little change for some, and considerable change for others.

### 3.6 Why do some participants’ decisional conflict scores rise?

Here, we contextualize decisional conflict scores with interview data. Participant P07's score increased 13 points from time 1 to 2. During his first interview, he described hoping for a cure and possibly staying off ART for the entire trial. At time 2, he reported negative physical and psychosocial impacts of several optional procedures and being disappointed by his rapid viral rebound. At the final interview, his score was low again; he reported doing well on ART and that he would make the same decision to participate.

Participant P08 had low decisional conflict scores at the first and second interviews, but his score rose 21 points in the third. Initially enthusiastic about RV411, he advised people to join because “this is the first place in the world that is running this project. We still do not have much information. We are like experimental mice. If the result is successful, it is good. But if it fails, we can restart [ART].” He expressed concern about seroconversion but felt protected by trial monitoring. Before the third interview, however, he learned he had seroconverted. He said, “I'm fine. It would have been positive one day,” and argued that researchers should continue with their work, even describing benefit to the failure of the trial: “[We have now learned that] this way doesn't work, so the likelihood that we will find the right way should increase.” However, he stated that if he had to do it again, he might not join: “It was interesting to participate, but it changed my anti‐HIV result to be positive.”

### 3.7 Why do some decliners have higher initial decisional conflict?

Like other decliners, the two with the highest initial scores [D04, D05] reported time commitment as a major factor in their decision. Interview data reveal that their health and HIV perceptions clearly influenced their assessment of personal risks. The decliner with the highest score [D05] had considerable difficulty adjusting to his HIV diagnosis and side effects of ART. Reflecting on recruitment to RV411, he wanted to stop taking ART, but, comparing himself with others doing well on ART, found reasons to decline: “I think that our bodies are not the same… . My health is not good.” Participating would “seem like I put my life at risk. I should think more carefully than other people.” The other decliner [D04] with a high initial score reported similar concerns about keeping his immune system healthy. After experiencing significant side effects from ART, he felt “normal” on treatment, in contrast to those participants who anticipated feeling “normal” when they stopped taking ART. He said he would be worried about going off ART without anything else, such as an experimental vaccine, to support him. In his final interview, he said he had wanted to join but was not ready.

## 4 Discussion

In this study of decision making for an HIV clinical trial of ATI 4, participants and decliners perceived that they made appropriate choices, with low overall decisional conflict. Given the ethical challenges associated with Phase 1 trials, and particularly Phase 1 HIV trials with individuals well‐controlled on ART, we critically appraised group and individual results for areas that could be improved, even for small subgroups of interviewees, and call for future research on these topics.

Decision making was initially more difficult for decliners than participants. The trial's time and travel requirements were seen as burdensome; most decliners were also unwilling to risk going off ART even within a carefully monitored clinical trial. Those with the highest initial decisional conflict scores were worried about their own health. The view of their bodies as not strong enough to withstand ATI contrasts sharply with statements by RV411 participants (and also participants in our prior study 3), who were eager to test their immune systems. The two decliners who worried about the risk of seroconversion mentioned feeling “selfish.” This may reflect concern about violating social norms of harmony and accommodation in interpersonal relations (called *Krengjai*) in the predominantly Buddhist Thai culture 3, 16, and might also underlie their higher decisional conflict scores. More research is needed to understand why decliners found the decision more difficult.

While decision making was initially described as fairly easy by most participants, our DMS design with longitudinal data collection allows for analysis of participants’ perceptions of their decision over time. Most participants’ decisional conflict scores were low and remained low over time, and although most described being satisfied with their decisions and their research contributions at the trial's end, two participants stated they would make a different choice about participation. Our study identified negative or unexpected consequences for some, including seroconversion, rapid viral rebound and adverse effects from optional procedures. While not required, the choice to undergo optional procedures raises important consent issues that require further study.

Nevertheless, most participants believed their choice to join RV411 was right, given what they knew at the time, and all reported being willing to consider participation in future SEARCH studies. It is not a simple matter to move from such data to normative conclusions about the ethical permissibility of “cure” research, either in this context or more broadly 10. It is possible to evaluate a decision given what was known at the time, and think it was the right or reasonable choice; alternatively, one can evaluate one's choices given what is known in hindsight, and wish one had chosen differently. Such judgements may indicate a degree of cognitive dissonance regarding whether it was the right choice overall. From a research ethics point of view, both forms of retrospection are normatively relevant.

Importantly, our empirical findings highlight the contributions of a nuanced, longitudinal exploration of trial perceptions. Even while most joiners endorsed overall decision satisfaction, interviewees reported both positives and negatives of trial participation, from which important lessons can be learned. The advantage of longitudinal data is the ability to detect both change and stability in decision making and to develop hypotheses about what is driving both.

Our findings about RV411 participants’ motivations extend results from our previous study of 12 participants in another SEARCH HIV remission trial with an experimental agent and ATI 3, and studies that ask PLWHIV about hypothetical decision making for similar trials 17, 18. We found that very early HIV diagnosis created a sense of having “special bodies” for research that offered the potential for reciprocal benefit to self and others, and that treatment interruption was perceived as both a potential personal risk and benefit; both themes were reproduced here. The RV411 data also support our prior study's finding that long‐standing, close relationships with the SEARCH team played an important role in decision making 3. This was reported in a positive frame by all participants except one [P07], whose description of initial decision making raises unease about whether the SEARCH staff were too influential, particularly as the “opportunity” to contribute to science might be construed as pressure, or possibly manipulation 19. Inherent conflicts of interest when recruitment occurs within long‐standing clinical and research relationships, such as those in SEARCH, have been well‐described 20, 21, 22. Managing such conflicts is possible when they are identified and when transparent recruitment and informed consent processes are employed. Table 4, created jointly with the trial investigators and the DMS team, summarizes implications of our findings for investigators conducting HIV remission trials and other similar research. The recommendations emerge directly from our study, and focus on ethical recruitment and support for both participants and decliners, as well as specific implications of conducting trials with individuals diagnosed and treated at the acute stage of infection.

**Table 4:** Points to consider for clinical trial investigators

1. Explore potential participants’ understanding and expectations during informed consent; identify areas where additional education and discussion are needed:
Explore sources of potential undue influence and emphasize voluntary nature of participation.
While allowing for optimism, facilitate a realistic understanding of anticipated outcomes.
Endeavour to elicit potential participants’ perceptions of anticipated benefits and harms because they may differ from those identified by the clinical trial team.
More forecasting about optional procedures may be warranted, reinforcing that they are optional, do not impact trial participation, and that may involve serial procedures
2. Understand motivations for and implications of declining participation:
Reinforce the appropriateness of diverse choices to decline participation, and if possible offer downstream research that may be more appealing to the individuals.
Be prepared to respond to psychosocial issues that emerge during the decision‐making process.
3. Consider specific implications of recruiting participants diagnosed/treated at the acute stage of HIV infection:
Acute status may be related to lower perceived risk of harm and higher perceived potential for benefit from trial participation.
Educate all potential participants about their serological status. Understand that seronegative status may confer special psychological and material benefits, and seropositive status may confer special risks, which vary by particular location/context.
4. Support participants during trial:
Those who are optimistic about analytic treatment interruption (ATI) may benefit from guidance that there are different ATI experiences and that they may have more anxiety than they expect during ATI.
Continue to discuss all potential risks of ATI, including transmission risk.
Confirm before each optional procedure that participant is still willing, reaffirm it is optional, and that procedures can be declined at any time without adversely affecting trial participation.
When negative outcomes such as viral rebound and seroconversion are experienced, be prepared to provide additional education and psychological support.

## 5 Conclusions

Although our portrait of eight participants and six decliners is exploratory, individual cases point to lessons for clinical trial investigators. These cases can help them prepare participants with realistic expectations about trial experiences and outcomes, and understand and support decliners in their decisions. We do not engage here in philosophical debate about the relative value of normative deliberation versus empirical data in making ethical judgements about the existence of HIV “cure” trials. Rather, we argue that in the context of an existing, ongoing area of clinical research, careful multi‐method assessments that are drawn over time can reveal how and why decisions are made, whether recruitment has been handled in the most ethical manner, and where potential concerns remain.

## Competing interests

Jintanat Ananworanich has participated in advisory meetings for ViiV Healthcare, Merck, AbbVie, Gilead and Roche. The remaining authors report no relevant conflicts of interest.

## Authors’ contributions

GEH: (1) made substantial contributions to the conception and design of the work, and to the analysis and interpretation of data for the work; (2) drafted and revised the work for important intellectual content; (3) gave final approval of the version to be published; and (4) agrees to be accountable for all aspects of the work in ensuring that questions related to the accuracy or integrity of any part of the work are appropriately investigated and resolved. MW: (1) made substantial contributions to the analysis and interpretation of data for the work; (2) drafted and revised the work for important intellectual content; (3) gave final approval of the version to be published; and (4) agrees to be accountable for all aspects of the work in ensuring that questions related to the accuracy or integrity of any part of the work are appropriately investigated and resolved. KM: (1) made substantial contributions to the analysis and interpretation of data for the work; (2) drafted and revised the work for important intellectual content; (3) gave final approval of the version to be published; and (4) agrees to be accountable for all aspects of the work in ensuring that questions related to the accuracy or integrity of any part of the work are appropriately investigated and resolved. RJC: (1) made substantial contributions to the conception and design of the work; and to the analysis and interpretation of data for the work; (2) drafted and revised the work for important intellectual content; (3) gave final approval of the version to be published; and (4) agrees to be accountable for all aspects of the work in ensuring that questions related to the accuracy or integrity of any part of the work are appropriately investigated and resolved. TJ: (1) made substantial contributions to the acquisition, analysis, and interpretation of data for the work; (2) revised the work critically for important intellectual content; (3) gave final approval of the version to be published; and (4) agrees to be accountable for all aspects of the work in ensuring that questions related to the accuracy or integrity of any part of the work are appropriately investigated and resolved. SI: (1) made substantial contributions to the conception and design of the work; and analysis and interpretation of data for the work; (2) drafted the work and revised it critically for important intellectual content; (3) gave final approval of the version to be published; and (4) agrees to be accountable for all aspects of the work in ensuring that questions related to the accuracy or integrity of any part of the work are appropriately investigated and resolved. NQO: (1) made substantial contributions to the analysis and interpretation of data for the work; (2) revised the work critically for important intellectual content; (3) gave final approval of the version to be published; and (4) agrees to be accountable for all aspects of the work in ensuring that questions related to the accuracy or integrity of any part of the work are appropriately investigated and resolved. DJC: (1) made substantial contributions to the design of the work; and interpretation of data for the work; (2) and revised the work critically for important intellectual content; (3) gave final approval of the version to be published; and (4) agrees to be accountable for all aspects of the work in ensuring that questions related to the accuracy or integrity of any part of the work are appropriately investigated and resolved. EK: (1) made substantial contributions to the design of the work; and to interpretation of data for the work; (2) revised the work critically for important intellectual content; (3) gave final approval of the version to be published; and (4) agrees to be accountable for all aspects of the work in ensuring that questions related to the accuracy or integrity of any part of the work are appropriately investigated and resolved. NP: (1) made substantial contributions to the interpretation of data for the work; (2) revised the work critically for important intellectual content; (3) gave final approval of the version to be published; and (4) agrees to be accountable for all aspects of the work in ensuring that questions related to the accuracy or integrity of any part of the work are appropriately investigated and resolved. JA: (1) made substantial contributions to the design of the work; and interpretation of data for the work; (2) revised the work critically for important intellectual content; (3) gave final approval of the version to be published; and (4) agrees to be accountable for all aspects of the work in ensuring that questions related to the accuracy or integrity of any part of the work are appropriately investigated and resolved. HLP: (1) made substantial contributions to the conception and design of the work; and analysis and interpretation of data for the work; (2) drafted the work and revised it critically for important intellectual content; (3) gave final approval of the version to be published; and (4) agrees to be accountable for all aspects of the work in ensuring that questions related to the accuracy or integrity of any part of the work are appropriately investigated and resolved.

## Appendix 1 O'Connor Decisional Conflict Scale Items

### 1.1

https://decisionaid.ohri.ca/docs/develop/User_Manuals/UM_Decisional_Conflict.pdf

Response categories range from strongly agree to strongly disagree

1. I know which options are available to me.
2. I know the benefits of each option.
3. I know the risks and side effects of each option.
4. I am clear about which benefits matter most to me.
5. I am clear about which risks and side effects matter most.
6. I am clear about which is more important to me (the benefits or the risks and side effects).
7. I have enough support from others to make a choice.
8. I am choosing without pressure from others.
9. I have enough advice to make a choice.
10. I am clear about the best choice for me.
11. I feel sure about what to choose.
12. This decision is easy for me to make.
13. I feel I have made an informed choice.
14. My decision shows what is important to me.
15. I expect to stick with my decision. [Note: Due to translation issues, we removed this item and adjusted the total scores to fall into the standard range of 0 to 100]
16. I am satisfied with my decision.
